# VIRdb: a comprehensive database for interactive analysis of genes/proteins involved in the pathogenesis of vitiligo

**DOI:** 10.7717/peerj.9119

**Published:** 2020-05-21

**Authors:** Priyansh Srivastava, Alakto Choudhury, Mehak Talwar, Sabyasachi Mohanty, Priyanka Narad, Abhishek Sengupta

**Affiliations:** Amity Institute of Biotechnology, Amity University, Uttar Pradesh, India

**Keywords:** Vitiligo, Database, Differential genes, Protein targets, Molecular docking, Natural compounds

## Abstract

Vitiligo is a chronic asymptomatic disorder affecting melanocytes from the basal layer of the epidermis which leads to a patchy loss of skin color. Even though it is one of the neglected disease conditions, people suffering from vitiligo are more prone to psychological disorders. As of now, various studies have been done in order to project auto-immune implications as the root cause. To understand the complexity of vitiligo, we propose the Vitiligo Information Resource (VIRdb) that integrates both the drug-target and systems approach to produce a comprehensive repository entirely devoted to vitiligo, along with curated information at both protein level and gene level along with potential therapeutics leads. These 25,041 natural compounds are curated from Natural Product Activity and Species Source Database. VIRdb is an attempt to accelerate the drug discovery process and laboratory trials for vitiligo through the computationally derived potential drugs. It is an exhaustive resource consisting of 129 differentially expressed genes, which are validated through gene ontology and pathway enrichment analysis. We also report 22 genes through enrichment analysis which are involved in the regulation of epithelial cell differentiation. At the protein level, 40 curated protein target molecules along with their natural hits that are derived through virtual screening. We also demonstrate the utility of the VIRdb by exploring the Protein–Protein Interaction Network and Gene–Gene Interaction Network of the target proteins and differentially expressed genes. For maintaining the quality and standard of the data in the VIRdb, the gold standard in bioinformatics toolkits like Cytoscape, Schrödinger’s GLIDE, along with the server installation of MATLAB, are used for generating results. VIRdb can be accessed through “http://www.vitiligoinfores.com/”.

## Introduction

Vitiligo is a chronic asymptomatic disorder affecting melanocytes from the basal layer of the epidermis which leads to a patchy loss of skin color (Rodrigues et al., 2017). It affects 0.5–2% of the world’s population (Ezzedine & Silverberg, 2016). The lesions can appear in different shapes and sizes on any visible part of the body (Tarlé et al., 2014). Even though it is one of the neglected diseases condition people suffering from vitiligo are more prone to psychological disorders and the risk of developing hearing problems (Ezzedine et al., 2015). The auto-immune inflammation theory remains to be the most acceptable, accounting for its development via an adaptive immune response (Boniface et al., 2017). According to biochemical theory, accumulation of Reactive Oxygen species (ROS) in the melanocytes remains the pioneer, for activation of CD8+ T cells against melanocytes (Wang, Li & Li, 2019; Deng et al., 2019). Previous studies have also reported that HIF-1-alpha, F2RL-1 and PIK3CB over-expression in CD8+ cells (Deng et al., 2019). HIF-1-alpha plays a crucial part in ROS accumulation response, whereas the F2RL-1 and PIK3CB are responsible for an immune response with CD8+ T cells (Deng et al., 2019). Due to loss of melanocytes in individuals affected by vitiligo, one can also expect an increased incidence of non-melanoma cancer and actinic keratose (Zhang et al., 2016). Arresting the progression of lesions and repigmentation of formed lesions remains to be a crucial strategy for treatment (Bishnoi & Parsad, 2018). AK-STAT inhibitors have demonstrated promising outcomes in the treatment of vitiligo, including repigmentation in topical applications. JAK inhibitor, tofacitinib and JAK-1,2 inhibitor, ruxolitinib, have been tested positive in causing repigmentation (Bishnoi & Parsad, 2018).

To understand the complexity of the vitiligo, we envisage a comprehensive online repository focusing on the systematically derived and manually curated potential protein targets along with their prospective hits derived from the natural compounds databases. To fully understand the genetic complexity of the vitiligo we also aim to present a genetic interaction network of the differentially expressed genes, in Lesional Vitiligo, Peri-Lesional Vitiligo, and Non-Lesional Vitiligo against healthy controls. The predicted differentially expressed genes across samples were then validated using gene enrichment analysis with Kyoto Encyclopedia of Gene and Genome (KEGG) and Gene Ontology (GO) (Kanehisa et al., 2020; The Gene Ontology Consortium, 2007). The Vitiligo Information Resource (VIRdb) systematically harbors all the information relating to vitiligo in terms of Potential protein targets and differentially expressed genes in a user-friendly database. As more than 50% of the approved drugs in DrugBank are natural compounds, therefore, we took the liberty of virtually screening all the natural compounds present in Natural Product Activity and Species Source Database (NPASS) against the potential protein targets (Wishart et al., 2017; Zeng et al., 2017). For maintaining, the quality and standard of the data in the VIRdb, the gold standard in bioinformatics toolkits like Cytoscape, Schrödinger’s GLIDE, along with the server installation of MATLAB are used (Shannon, 2003; Schrödinger, 2020; MATLAB, 2019). The screened library can be used to carry wet-lab experiments based on potential hits which would thereby lead to considerably less consumption of time and other resources to carry out the protocol for drug development. We envision that VIRdb will be pertinent for the researchers and clinicians engaged in drug development for vitiligo.

## Methods

### Microarray gene expression analysis

Gene Expression Omnibus was manually queried using “(“vitiligo” (MeSH Terms) OR vitiligo (All Fields)) AND (“humans” (MeSH Terms) OR “*Homo sapiens*” (Organism) OR homo sapiens (All Fields))”. The returned results were manually curated for microarray experiments having healthy controls against affected individuals. We selected GSE65127 as it comprises of 10 healthy controls against 10 conditions (Lesional, Peri-lesional, Non-lesional). The CEL files of 40 samples having expression data of Lesional, Peri-lesional, Non-lesional against healthy control on Affymetrix GPL570 platform were isolated (Clough & Barrett, 2016; Regazzetti et al., 2015) data. Combinations of {Healthy vs (Lesional, Peri-lesional, Non-lesional)} were processed using the server installation of MATLAB (2019) by applying the GC Robust Multichip Averaging (GCRMA) method with Empirical Bayes (MATLAB, 2019; Wu et al., 2004). GCRMA empirical Bayes is the posterior mean estimate which is calculated through *S*, that represents a quantity proportional to the concentration of transcripts in the hybridization mixture (Eq. 1). After the raw reads from CEL files are converted to expression data through GCRMA, the probe sets with un-annotated gene symbols were dropped programmatically. A *t*-test is performed with each gene expression value to study the relevant changes in the expression values. False Discovery Rate (FDR) for each test situation was computed using Storey–Tibshirani procedure (Storey & Tibshirani, 2003). To compute the biological expression significance, −log10 of *p*-values was calculated and plotted against log fold change (2 folds).

(1) $$s = E\left[ {s|S \gt 0,{\rm PM},{\rm MM}} \right], \quad \; {\rm with}\ s = {\rm log}\left( S \right)$$

### Gene–gene interaction network construction

For designing the gene–gene interaction network for differentially expressed genes, GeneMania plugin from Cytoscape was used (Shannon, 2003; Mostafavi et al., 2020). Initially, 3 distinct networks for differentially expressed genes (Expressed in lesional, Expressed in non-lesional, and Expressed in peri-lesional) were made. After that, all the networks are merged, with common interacting gene nodes in the Cytoscape environment (Shannon, 2003). The tangential nodes were manually deleted and only the highly connected nodes were taken for further analysis. The predicted co-expressed genes from GeneMania interaction network were also taken for enrichment analysis (Mostafavi et al., 2020).

### Gene Ontology (GO) and pathway analysis

ShinyGO server was used for the pathway enrichment analysis of the single expression conditions (Lesional, Peri-Lesional and Non-lesional, each distinctly) and unique differentially expressed genes (significant expression at least once across the conditions) and predicted GGI genes from GeneMania (Mostafavi et al., 2020; Ge, Jung & Yao, 2019). The differentially expressed genes were enriched in Kyoto Encyclopedia of Gene and Genome database (KEGG) (Kanehisa et al., 2020). The false discovery rate of 0.01 was used as the cut off to elute out the most significant pathways. For GO enrichment analysis the GO database was recruited from the ShinyGO server and GO enrichment analysis was functioned with the false discovery rate of <0.01 (The Gene Ontology Consortium, 2007; Mostafavi et al., 2020).

### Biological data collection

The Pubmed Engine was queried using “((Vitiligo) AND (Proteins)) AND (Protein–Protein Interaction)” for retrieval of literature relating to the protein targets (Motschall & Falck-Ytter, 2005). The query returned 4 abstracts, out of which study that includes the systemic approach for the construction of Protein–Protein Interaction Network (PPI) was taken into consideration (Malhotra et al., 2017). PPI network of 215 proteins was revitalized using Cytoscape 3.7 and String Database (Shannon, 2003; Mering, 2003; Malhotra et al., 2017). A total of 50 protein targets were eluted out of the set of reported 64 vitiligo core proteins in the study (Malhotra et al., 2017). The sub-network of 50 proteins was created manually based on coherence of centrality among 64 nodes (Malhotra et al., 2017). Finally considering the on the availability of X-ray crystallographic structures in Protein Data Bank 40 protein crystal structures were taken for molecular docking out of 50 nodes (Appendix I) (Berman, 2000; Malhotra et al., 2017). All the potential protein targets were annotated manually with their respective UniProt ID, PDB ID, STRING ID, KEGG ID, encoding Gene Symbol and target active amino acids from the scientific literature (Kanehisa et al., 2020; Mering, 2003; Berman, 2000; The UniProt Consortium, 2016). Highest-resolution structures were manually curated and confiscated for Molecular docking studies for the protein structures that comprises multiple deposits in the Protein Data Bank (Wlodawer et al., 2013). For identification of the active amino acids, each PDB profile was manually curated in reference to its literature (Appendix I).

### Virtual screening against NPASS

As more than 50% of the approved drugs in DrugBank are natural compounds, therefore, we took the liberty of virtually screening all the natural compounds present in Natural Product Activity and Species Source Database (NPASS) against the potential protein targets (Wishart et al., 2017; Zeng et al., 2017). The NPASS database consists of 25,041 natural compounds (Zeng et al., 2017). Every structure was neutralized at the physiological pH and a library of 36,229 potential ligands was created including the stereoisomers and tautomers (Srivastava, Pal & Misra, 2018). Target protein structures files were prepared using Schrödinger Protein Preparation Wizard by removing excess waters and were minimized using OPLS 2005 force field (Clark et al., 2019; Sweere & Fraaije, 2017). Receptor grid of dimensions of 15 × 15 × 15 angstroms was generated around the active amino acids. Glide HTVS pipeline was used to carry out virtual screening of the natural compound library to each target protein. Ligands were sorted and ranked based on the Glide score as it is empirical of various computed scores and is also quite accurate in wet-lab experimentation (Greenwood et al., 2010).

### Architecture and design

**Figure 1:**
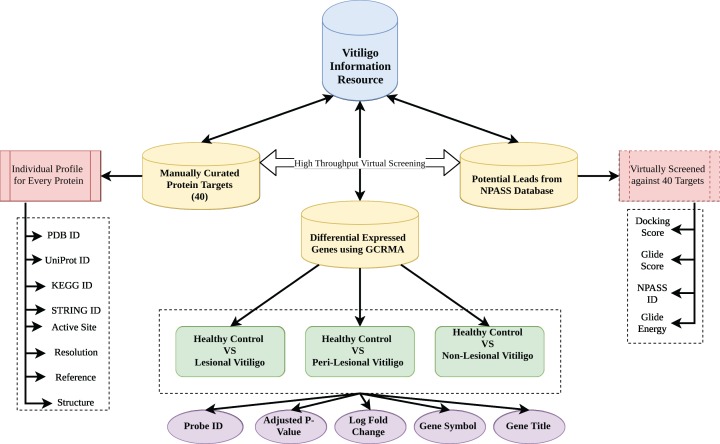
Database architecture. Architecture of VIR database.

Vitiligo Information Resource was formulated using Apache HTTP server on Linux Platform. MySQL was used to store the information as it offers fast and easy query processing because of its RDBMS architecture (Mathur et al., 2016). HTML 5 and Cascading Style Sheets were used to design the front-end interface of the VIRdb. PHP 7 was used for maintaining the dynamic connectivity between the front-end and back-end. Each entry in the database was connected to their parent database via html anchors. The complete architectural schema of vitiligo information resource db is depicted in Fig. 1.

## Results

### Differentially expressed genes

**Figure 2:**
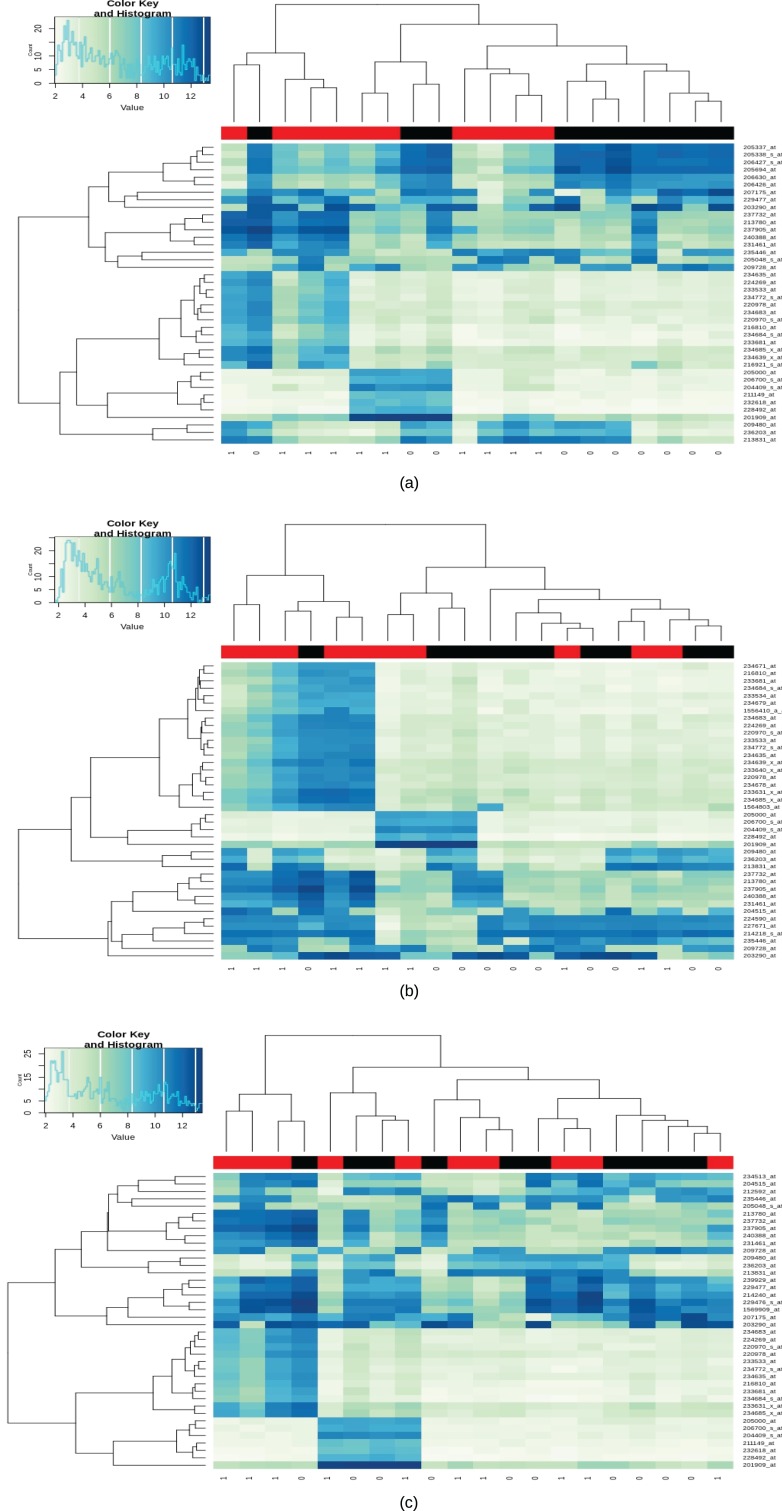
Heatmap for lesional, non-lesional and peri-lesional conditions. Heatmap of differentially expressed gene expression values with black bars representing the Healthy/control samples and red being the case/diseased samples; (A) heatmap of differentially expressed gene expression values in lesional vitiligo against Healthy controls; (B) heatmap of differentially expressed gene expression values in non-lesional vitiligo against healthy controls; (C) heatmap of differentially expressed gene expression values in peri-lesional vitiligo against healthy controls.

A total of 123 differential genes for lesional vitiligo against healthy controls. Out of 123 genes, 52 genes were predicted to be as under-expressed and 70 genes were predicted to be over-expressed in accordance with the LogFc filter (Appendix II; Fig. 2A). In the case of Non-Lesional vs healthy control, a total of 27 upregulated differential genes were identified (Appendix III; Fig. 2B). For Peri-Lesional vitiligo, a total of 32 upregulated genes were curated (Appendix IV; Fig. 2C). Upon, a union of all the sets (Differential Genes, Lesional, Non-Lesional, and Peri-Lesional) as a total of 129 differential genes have been curated. Out of which 52 were under-expressed and 76 were over-expressed (Appendix V).

### GO and pathway enrichment analysis

The DCT, EDNRB, TYR, TYRP1, and WNT6 that were from the set of 129 unique differentially expressed genes across the samples were enriched in Melanogenesis pathway with an FDR of 0.0038 in KEGG (Kanehisa et al., 2020) (Appendix VI). Upon GO enrichment 6 genes (GPR143, OCA2, TYR, DCT, TYRP1, SLC45A2) were enriched in Melanosome membrane (Cellular Compartment), 7 genes (TYR, DCT, TYRP1, OCA2, SLC45A2, PMEL, CDH3) were enriched in Melanin metabolic process (Biological Process) (The Gene Ontology Consortium, 2007) (Appendix VII). Surprisingly, 2 genes were enriched with monophenol monooxygenase activity (molecular function) and 2 genes were enriched in oxidoreductase activity (molecular function), which could account for the oxidative stress-induced melanocyte degradation (Wang, Li & Li, 2019; Deng et al., 2019) (Appendix VII) (For enrichment analysis of the single expression conditions (Lesional, Peri-Lesional and Non-lesional, each distinctly) see Supplements 1–3).

### Gene–gene interaction network

We upvote 22 genes that were predicted while construction of common network (ACYP1, APLP2, ARNTL, CEBPG, CPEB2, DIP2A, HNRNPA3, HNRNPA3P1, HTATSF1, JKAMP, MAFG, MFN2, MKL1, ORC2, PAGR1, PDPK1, PEX5, PRKX, PRKY, TLE4, TMEM70, USP47) with GeneMania (Shannon, 2003, Fig. 3; Appendix VIII). Upon gene enrichment analysis of these 22 genes with GO db MAFG, PRKX, ARNTL were enriched in Regulation of epithelial cell differentiation as the biological process (The Gene Ontology Consortium, 2007). These genes were never reported in the literature for these types of vitiligo therefore, might be relevant in understanding the molecular aspects of vitiligo.

**Figure 3:**
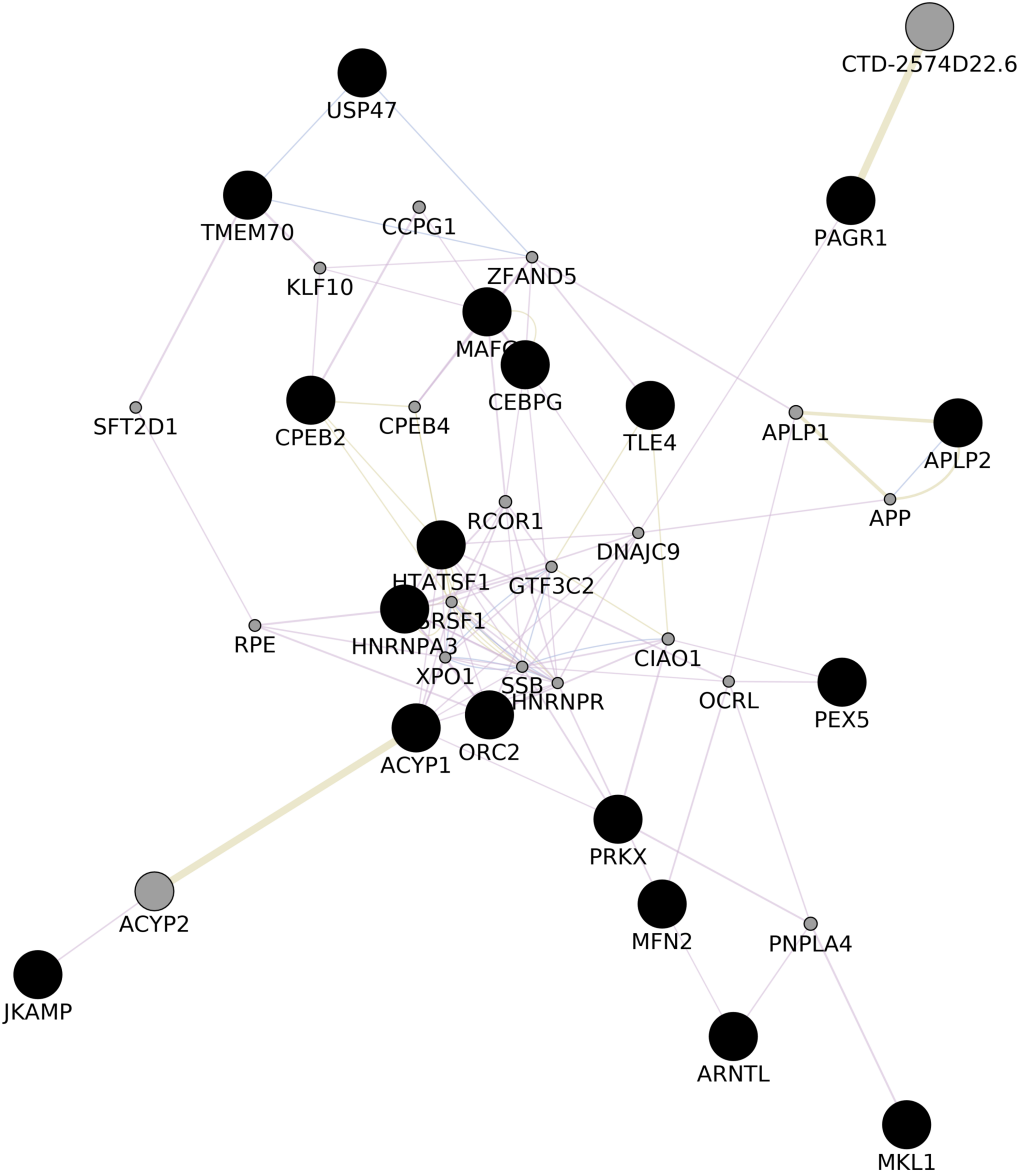
GGI Network. Gene–gene Interaction network of the predicted set of differential genes in lesional, non-lesional, and peri-lesional condition through the set of 129 unique genes; co-expressed genes are shown in gray nodes; constructed using Cytoscape 3.7 (Shannon, 2003).

### Data browsing and web interface

**Figure 4:**
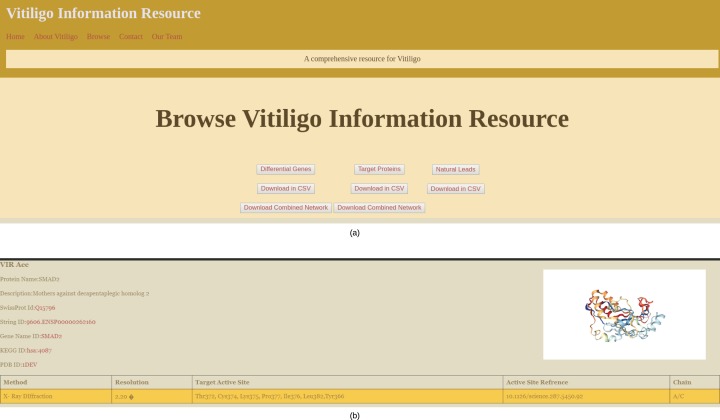
VIRdb Interface. (A) Browsing Interface of VIRdb and (B) individual protein profile.

The main browsing window of the VIRdb holds the links to Target proteins, Potential hits and differential genes (Fig. 4A). The end-user can either browse any of the sections or can directly download the data in CSV file format. The network files are only available for download and can be downloaded in SIF of the Cytoscape 3.7. The target protein toggle directs the user to the comprehensive list of proteins that were used as targets for the molecular docking studies (Shannon, 2003). The other two toggles of potential hits and differential genes direct the user towards the dynamic tables where the user can select from the types of vitiligo and target specific drugs. The target proteins have individual profiles as shown in Fig. 4B, which comprises their active site residue references along with the potential hits against it. The identifiers (STRING ID, KEGG ID, PDB Id, SwissProt Id) of the target proteins are connected to their respective databases through the HTML anchors (Kanehisa et al., 2020; Mering, 2003; Berman, 2000; The UniProt Consortium, 2016). The encoding genes for the proteins are connected to the GeneCards database for maintaining the quality of data (Safran et al., 2010). The user can directly download the molecular docking results (NPASS ids, Docking Scores, Glide gscores, and Glide energy) in CSV file format from the individual protein profiles for further analysis (Zeng et al., 2017). The active site reference of the protein is depicted in a tabular format depicting the amino residue name, chain and position in the sequence. Potential hits can be browsed through dynamic tables and frames. The user can select the protein’s PDB identifier from below the navigation pane and can dynamically see the tailored results for the potential hits against them. The tables contain information about the NPASS db identifier, Glide Gscore, Docking Score and Glide energy, that correlates to the binding affinity of the ligand with the target. Each NPASS identifier is connected to the NPASS db entry of the respective compound (Zeng et al., 2017). The user can also download the results from this page in CSV files formats for further analysis. Differential genes that are predicted using MATLAB (2019) are also projected in a dynamic fashion. The end-user can see the genes by selecting the appropriate toggle from below the navigation bar. The table consists of Probe Ids, LogFc, Gene Symbol, Adjusted *p*-values and gene descriptions. Each gene symbol is connected to the GeneCard entry through HTML anchors (Safran et al., 2010). LogFc values with negative signs denote the downregulated genes while the positive ones account for the upregulation. The probe IDs of the genes are connected to Harvard’s Gene Enrichment Profiler (Bateman et al., 2014).

### Discussion and community impact

As per the results of KEGG enrichment analysis, the differential genes were enriched in the pathway of Melanogenesis. WNT6 being one of the enriched genes has been previously reported for vitiligo therapeutics and wound healing through melanocyte stem cell differentiation (Yamada et al., 2013; Sun et al., 2018). Moreover, DCT, TYR, EDNRB and TYRP1 have also been reported previously playing a vital role in vitiligo (Salinas-Santander et al., 2018; Jin et al., 2010; Takeo et al., 2016). Therefore, other differential genes which were enriched along with WNT6, DCT, TYR, EDNRB and TYRP1 by GO could provide more insights on vitiligo prognosis (Appendix VII). We also report ACYP1, APLP2, ARNTL, CEBPG, CPEB2, DIP2A, HNRNPA3, HNRNPA3P1, HTATSF1, JKAMP, MAFG, MFN2, MKL1, ORC2, PAGR1, PDPK1, PEX5, PRKX, PRKY, TLE4, TMEM70, USP47 for these types of vitiligo (Lesional, Non-lesional, and Peri-Lesional) therefore might be relevant in understanding the molecular aspects of vitiligo. VIRdb, integrates both drug-target and systems approach to produce a comprehensive repository entirely devoted to Vitiligo. Having curated information at both protein level and gene level along with potential therapeutics hits makes VIRdb first and one of its kind disease-specific databases in the scientific community. VIRdb holds manually curated protein targets that are involved in the vitiligo based on the systems approach, these protein target structure files are minimized and optimized using OPLS 2005 force fields and can be downloaded from the database for carrying out in-silico analysis. VIR also offers a complete profile for these target protein having links to all major databases (KEGG db, STRING db, GeneCards db, SwissProt db, NPASS db) making it a cross-functional database as well (Kanehisa et al., 2020; Mering, 2003; The UniProt Consortium, 2016; Safran et al., 2010).

## Conclusion

Lesional Vitiligo, Peri-Lesional vitiligo, and Non-Lesional vitiligo being the most common types of vitiligo have been thoroughly studied for differentially expressed genes and are descriptively integrated into the compendium of VIRdb. All of which are validated through KEGG and GO db, having pathways in functions in melanogenesis (Kanehisa et al., 2020; The Gene Ontology Consortium, 2007). It is also the home for the potentially active natural compounds that are virtually screened against the potential protein targets. These compounds are curated from NPASS db (Zeng et al., 2017). It is an attempt to accelerate the drug discovery process and laboratory trials for vitiligo. We will update VIRdb iteratively at regular intervals in order to cover the domain of vitiligo as a diseased condition. We also provide users with an option to submit new data. Moreover, we will validate each new entry before its incorporation for maintaining a high level of quality of data. In future various improvizations on the VIRdb could be done like the inclusion of various other chemical databases like ZINC, DrugBank and PubChem (Wishart et al., 2017; Irwin & Shoichet, 2005; Kim et al., 2015).

## Supplemental Information

10.7717/peerj.9119/supp-1 Supplemental Information 1 Appendix 1. All protein Targets

10.7717/peerj.9119/supp-2 Supplemental Information 2 Appendix 2. Differentially Expressed Genes for Lesional Vitiligio

10.7717/peerj.9119/supp-3 Supplemental Information 3 Appendix 3. Differentially expressed genes for Non-Lesiional Vitiligo

10.7717/peerj.9119/supp-4 Supplemental Information 4 Appendix 4. Diffferentially expressed genes for Peri-Lesional Vitiligo

10.7717/peerj.9119/supp-5 Supplemental Information 5 Appendix 5. Union of Differentially Expressed gene across all 3 conditions (lesional, non-lesion, peri-lesional)

10.7717/peerj.9119/supp-6 Supplemental Information 6 Appendix 6. KEGG enrichment analysis of the union of set of genes across 3 conditions (lesional, non-lesion, peri-lesional)

10.7717/peerj.9119/supp-7 Supplemental Information 7 Appendix 7. GO enrichment analysis of the union of set of genes across 3 conditions (lesional, non-lesion, peri-lesional)

10.7717/peerj.9119/supp-8 Supplemental Information 8 Appendix 8. 22 Predicted genes via GeneMania plugin of Cytoscape

10.7717/peerj.9119/supp-9 Supplemental Information 9 GO Enrichment Results of DEG of Lesional vs. Healthy Condition.

10.7717/peerj.9119/supp-10 Supplemental Information 10 GO Enrichment Results of DEG of Non-Lesional vs. Healthy Condition.

10.7717/peerj.9119/supp-11 Supplemental Information 11 GO Enrichment Results of DEG of Peri-Lesional vs. Healthy Condition.

## Additional Information and Declarations

### Data Availability

The following information was supplied regarding data availability: The analysis results are available as Supplemental Files. The database is available at https://vitiligoinfores.com/.
